# Ethyl 2-amino-4-(4-bromo­phen­yl)-6-meth­oxy-4*H*-benzo[*h*]chromene-3-carboxyl­ate

**DOI:** 10.1107/S160053681300490X

**Published:** 2013-02-23

**Authors:** Ahmed M. El-Agrody, Ahmed M. Fouda, Mohamed A. Al-Omar, Abd El-Galil E. Amr, Seik Weng Ng, Edward R. T. Tiekink

**Affiliations:** a Chemistry Department, Faculty of Science, King Khalid University, Abha 61413, PO Box 9004, Saudi Arabia; b Chemistry Department, Faculty of Science, Al-Azhar University, Nasr City, Cairo, 11884, Egypt; c Department of Pharmaceutical Chemistry, College of Pharmacy, King Saud University, Riyadh 11451, Saudi Arabia; d Drug Exploration & Development Chair (DEDC), College of Pharmacy, King Saud University, Riyadh 11451, Saudi Arabia; e Applied Organic Chemistry Department, National Research Center, Dokki 12622, Cairo, Egypt; f Department of Chemistry, University of Malaya, 50603 Kuala Lumpur, Malaysia; g Chemistry Department, Faculty of Science, King Abdulaziz University, PO Box 80203 Jeddah, Saudi Arabia

## Abstract

In the title compound, C_23_H_20_BrNO_4_, the pyran ring has a flattened boat conformation with the O and methine C atoms lying to one side of the plane [0.160 (5) and 0.256 (6) Å, respectively] defined by the remaining atoms. Nevertheless, the 4*H*-benzo[*h*]chromene ring system approximates a plane (r.m.s. deviation = 0.116 Å) with the bromo­benzene ring almost perpendicular [dihedral angle = 83.27 (16)°] and the ester group coplanar [C—C—C—O = 3.4 (5)°]; the meth­oxy substituent is also coplanar [C—O—C—C = 174.5 (3)°]. In addition to an intra­molecular N—H⋯O(ester carbon­yl) hydrogen bond, the ester carbonyl O atom also forms an inter­molecular N—H⋯O hydrogen bond with the second amine H atom, generating a zigzag supra­molecular chain along the *c* axis in the crystal packing. The chains are linked into layers in the *bc* plane by N—H⋯Br hydrogen bonds, and these layers are consolidated into a three-dimensional architecture by C—H⋯π inter­actions.

## Related literature

For background to the pharmaceutical activity of 4*H*-chromene and its derivatives, see: Abd-El-Aziz *et al.* (2004 ▶, 2007 ▶); Kemnitzer *et al.* (2007 ▶); Alvey *et al.* (2009 ▶). For the isostructural 4-fluoro analogue, see: El-Agrody *et al.* (2012 ▶).
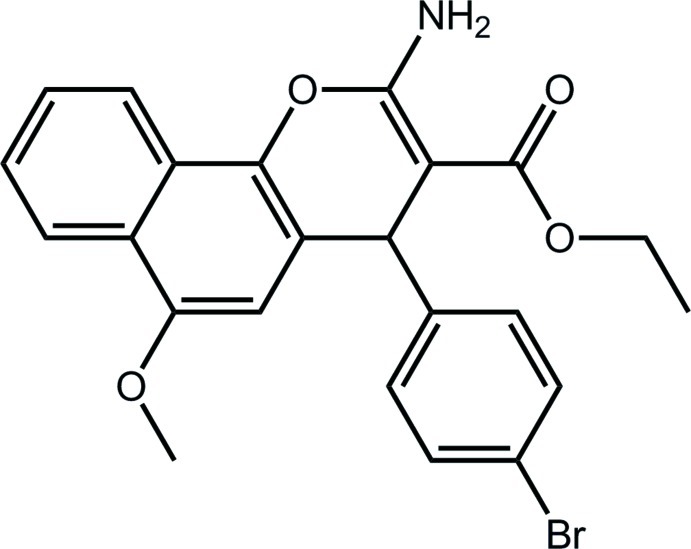


## Experimental

### 

#### Crystal data

C_23_H_20_BrNO_4_
*M* *_r_* = 454.31
Monoclinic, 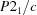

*a* = 13.1543 (14) Å
*b* = 16.8110 (18) Å
*c* = 9.3672 (12) Å
β = 96.628 (10)°
*V* = 2057.6 (4) Å^3^
*Z* = 4
Mo *K*α radiation
μ = 2.03 mm^−1^
*T* = 295 K
0.30 × 0.20 × 0.03 mm

#### Data collection

Agilent SuperNova Dual diffractometer with an Atlas detector
Absorption correction: multi-scan (*CrysAlis PRO*; Agilent, 2011 ▶) *T*_min_ = 0.828, *T*_max_ = 1.000
12041 measured reflections
4740 independent reflections
2533 reflections with *I* > 2σ(*I*)
*R*_int_ = 0.054

#### Refinement

*R*[*F* ^2^ > 2σ(*F* ^2^)] = 0.060
*wR*(*F* ^2^) = 0.164
*S* = 1.02
4740 reflections
270 parameters
2 restraints
H atoms treated by a mixture of independent and constrained refinement
Δρ_max_ = 0.91 e Å^−3^
Δρ_min_ = −0.90 e Å^−3^

### 

Data collection: *CrysAlis PRO* (Agilent, 2011 ▶); cell refinement: *CrysAlis PRO*; data reduction: *CrysAlis PRO*; program(s) used to solve structure: *SHELXS97* (Sheldrick, 2008 ▶); program(s) used to refine structure: *SHELXL97* (Sheldrick, 2008 ▶); molecular graphics: *ORTEP-3 for Windows* (Farrugia, 2012 ▶) and *DIAMOND* (Brandenburg, 2006 ▶); software used to prepare material for publication: *publCIF* (Westrip, 2010 ▶).

## Supplementary Material

Crystal structure: contains datablock(s) global, I. DOI: 10.1107/S160053681300490X/hg5295sup1.cif

Structure factors: contains datablock(s) I. DOI: 10.1107/S160053681300490X/hg5295Isup2.hkl

Supplementary material file. DOI: 10.1107/S160053681300490X/hg5295Isup3.cml

Additional supplementary materials:  crystallographic information; 3D view; checkCIF report

## Figures and Tables

**Table 1:** Hydrogen-bond geometry (Å, °) *Cg*1, *Cg*2 and *Cg*3 are the centroids of the C1,C2,C7–C10, C17–C22 and C2–C7 rings, respectively.

*D*—H⋯*A*	*D*—H	H⋯*A*	*D*⋯*A*	*D*—H⋯*A*
N1—H2⋯O2	0.88 (1)	2.09 (5)	2.744 (5)	131 (5)
N1—H1⋯O2^i^	0.88 (1)	2.22 (2)	3.075 (5)	163 (3)
N1—H2⋯Br1^ii^	0.88 (4)	2.76 (4)	3.547 (4)	149 (5)
C4—H4⋯*Cg*1^i^	0.93	2.90	3.673 (5)	142
C6—H6⋯*Cg*2^iii^	0.93	2.98	3.743 (5)	140
C23—H23*C*⋯*Cg*3^iii^	0.96	2.70	3.593 (5)	154

Symmetry codes: (i) 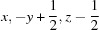; (ii) 
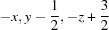
; (iii) 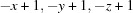.

**Fig. 1.**
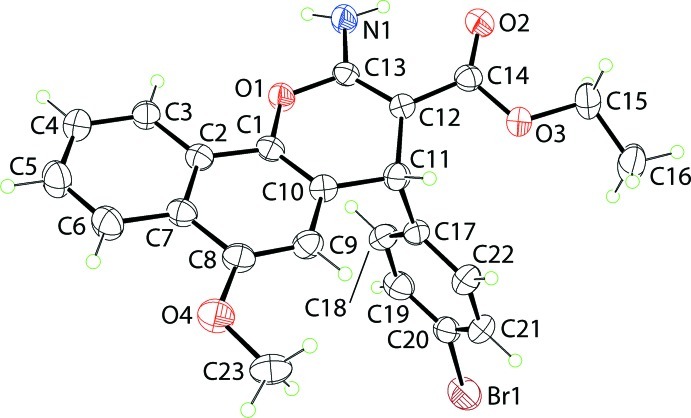
The molecular structure of (I) showing the atom-labelling scheme and displacement ellipsoids at the 35% probability level.

**Fig. 2.**
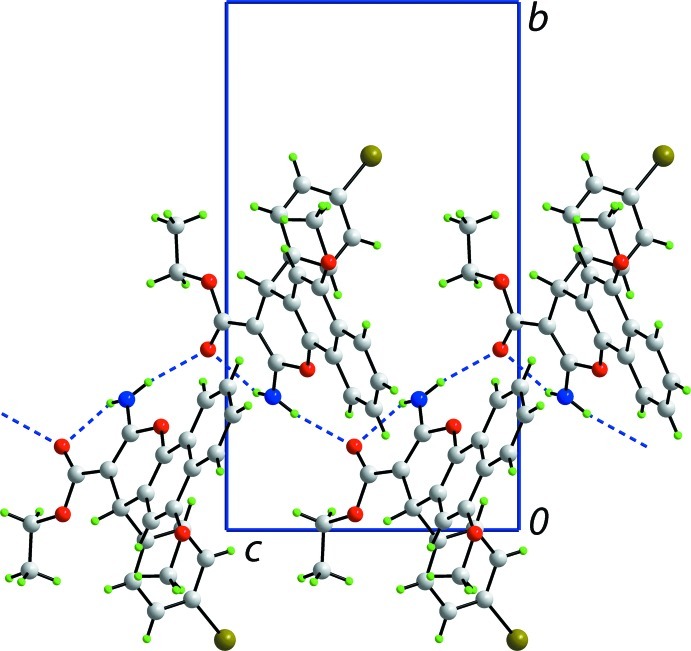
A view of the zigzag supramolecular chain along the c axis in (I) sustained by N—H···N hydrogen bonds, shown as blue dashed lines.

**Fig. 3.**
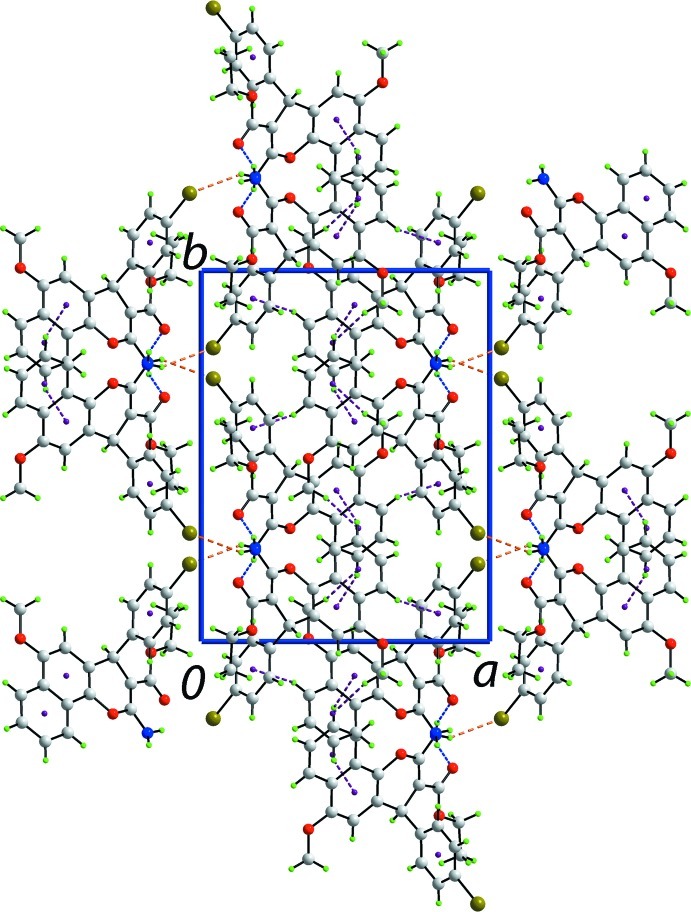
view in projection down the c axis of the crystal packing in (I). The N—H···N, N—H···Br and C—H···π interactions are shown as blue, orange and purple dashed lines, respectively.

## supplementary crystallographic information

### Comment

4*H*-Chromene and its derivatives are biologically interesting compounds known for their anti-microbial, anti-fungal and other pharmaceutical activities (Alvey *et al.*, 2009; Kemnitzer *et al.*, 2007). In continuation of on-going interest in the chemical and pharmacological properties of 4*H*-chromene and fused 4*H*-chromene derivatives (Abd-El-Aziz *et al.*, 2004; Abd-El-Aziz *et al.*, 2007), the crystal structure of the title compound, (I), is described herein.

The molecular structure of (I), Fig. 1, is isostructural to the recently reported 4-fluoro analogue (El-Agrody *et al.*, 2012). The pyran ring has a flattened boat conformation with the O1 and C11 atoms lying 0.160 (5) and 0.256 (6) Å, respectively, out of the plane defined by the four remaining atoms (r.m.s. deviation = 0.0174 Å). Overall, the 4*H*-benzo[*h*]chromene ring system is approximately planar with the r.m.s. deviation of the 14 non-hydrogen atoms being 0.116 Å. The bromobenzene ring is almost perpendicular to this plane, forming a dihedral angle of 83.27 (16)°. By contrast, the ester group, with an *anti* conformation [C14—O3—C15—C16 torsion angle = -166.9 (4)°], is co-planar [C11—C12—C14—O3 = 3.4 (5)°] due, in part, to an intramolecular N—H···O2 hydrogen bond, Table 1. The methoxy [C23—O4—C7—C7 = 174.5 (3)°] substituent is also co-planar to the ring to which it is attached.

Zigzag (glide symmetry) supramolecular chains along the *c* axis feature in the crystal packing owing to N—H···O2 hydrogen bonding, Fig. 2 and Table 1. Chains are linked into layers in the *bc* plane by N—H···Br hydrogen bonds involving the H atom involved in the intramolecular interaction to the O2 atom, Table 1. A consequence of this interaction is that the Br1 and O2 atoms are brought into close proximity, *i.e*. Br1···O2^i^ = 3.179 (3) Å [*i*: -*x*, 1/2 + *y*, 3/2 - *z*]. The three-dimensional architecture is consolidated by C—H···π interactions, Fig. 3 and Table 1.

### Experimental

A solution of 4-methoxy-1-naphthol (0.01 mol) in EtOH (30 ml) was treated with ethyl α-cyano-*p*-bromocinnamate (0.01 mol) and piperidine (0.5 ml). The reaction mixture was heated until complete precipitation occurred after 2 h. The solid product was collected by filtration and recrystallized from ethanol to give (I); *M*.pt: 438–439 K.

### Refinement

The C-bound H atoms were geometrically placed (C—H = 0.93–0.98 Å) and refined as riding with *U_iso_*(H) = 1.2–1.5*U_eq_*(C). The N-bound-H atom was refined with the distance restraint N—H = 0.88±0.01 Å and free *U*_iso_.

### Crystal data

**Table:**

C_23_H_20_BrNO_4_	*F*(000) = 928
*M**_r_* = 454.31	*D*_x_ = 1.467 Mg m^−^^3^
Monoclinic, *P*2_1_/*c*	Mo *K*α radiation, λ = 0.71073 Å
Hall symbol: -P 2ybc	Cell parameters from 1932 reflections
*a* = 13.1543 (14) Å	θ = 2.8–27.5°
*b* = 16.8110 (18) Å	µ = 2.03 mm^−^^1^
*c* = 9.3672 (12) Å	*T* = 295 K
β = 96.628 (10)°	Plate, light-orange
*V* = 2057.6 (4) Å^3^	0.30 × 0.20 × 0.03 mm
*Z* = 4	

### Data collection

**Table:**

Agilent SuperNova Dual diffractometer with an Atlas detector	4740 independent reflections
Radiation source: SuperNova (Mo) X-ray Source	2533 reflections with *I* > 2σ(*I*)
Mirror monochromator	*R*_int_ = 0.054
Detector resolution: 10.4041 pixels mm^-1^	θ_max_ = 27.6°, θ_min_ = 2.9°
ω scan	*h* = −16→17
Absorption correction: multi-scan (*CrysAlis PRO*; Agilent, 2011)	*k* = −21→21
*T*_min_ = 0.828, *T*_max_ = 1.000	*l* = −7→12
12041 measured reflections	

### Refinement

**Table:**

Refinement on *F*^2^	Primary atom site location: structure-invariant direct methods
Least-squares matrix: full	Secondary atom site location: difference Fourier map
*R*[*F*^2^ > 2σ(*F*^2^)] = 0.060	Hydrogen site location: inferred from neighbouring sites
*wR*(*F*^2^) = 0.164	H atoms treated by a mixture of independent and constrained refinement
*S* = 1.02	*w* = 1/[σ^2^(*F*_o_^2^) + (0.0575*P*)^2^ + 0.7996*P*] where *P* = (*F*_o_^2^ + 2*F*_c_^2^)/3
4740 reflections	(Δ/σ)_max_ = 0.001
270 parameters	Δρ_max_ = 0.91 e Å^−^^3^
2 restraints	Δρ_min_ = −0.90 e Å^−^^3^

### Special details

**Table:**

Geometry. All e.s.d.'s (except the e.s.d. in the dihedral angle between two l.s. planes) are estimated using the full covariance matrix. The cell e.s.d.'s are taken into account individually in the estimation of e.s.d.'s in distances, angles and torsion angles; correlations between e.s.d.'s in cell parameters are only used when they are defined by crystal symmetry. An approximate (isotropic) treatment of cell e.s.d.'s is used for estimating e.s.d.'s involving l.s. planes.
Refinement. Refinement of *F*^2^ against ALL reflections. The weighted *R*-factor *wR* and goodness of fit *S* are based on *F*^2^, conventional *R*-factors *R* are based on *F*, with *F* set to zero for negative *F*^2^. The threshold expression of *F*^2^ > σ(*F*^2^) is used only for calculating *R*-factors(gt) *etc*. and is not relevant to the choice of reflections for refinement. *R*-factors based on *F*^2^ are statistically about twice as large as those based on *F*, and *R*- factors based on ALL data will be even larger.

### Fractional atomic coordinates and isotropic or equivalent isotropic displacement parameters (Å^2^)

**Table:**

	*x*	*y*	*z*	*U*_iso_*/*U*_eq_	
Br1	0.04105 (4)	0.70821 (4)	0.50571 (7)	0.0900 (3)	
O1	0.30720 (18)	0.30429 (15)	0.7238 (3)	0.0464 (7)	
O2	0.1283 (2)	0.34197 (17)	1.0635 (3)	0.0539 (7)	
O3	0.1749 (2)	0.47024 (17)	1.0602 (3)	0.0558 (7)	
O4	0.6285 (2)	0.50690 (18)	0.6512 (3)	0.0634 (8)	
N1	0.1869 (3)	0.2527 (2)	0.8418 (4)	0.0538 (9)	
H2	0.138 (3)	0.260 (4)	0.897 (5)	0.11 (2)*	
H1	0.182 (3)	0.2203 (18)	0.768 (3)	0.044 (12)*	
C1	0.3874 (3)	0.3580 (2)	0.7136 (4)	0.0397 (9)	
C2	0.4654 (3)	0.3302 (2)	0.6337 (4)	0.0399 (9)	
C3	0.4637 (3)	0.2537 (2)	0.5709 (4)	0.0447 (9)	
H3	0.4095	0.2194	0.5807	0.054*	
C4	0.5416 (3)	0.2300 (3)	0.4959 (4)	0.0533 (11)	
H4	0.5404	0.1792	0.4562	0.064*	
C5	0.6228 (3)	0.2812 (3)	0.4783 (5)	0.0613 (12)	
H5	0.6752	0.2644	0.4267	0.074*	
C6	0.6260 (3)	0.3553 (3)	0.5359 (5)	0.0579 (12)	
H6	0.6802	0.3889	0.5223	0.069*	
C7	0.5480 (3)	0.3824 (2)	0.6167 (4)	0.0436 (9)	
C8	0.5483 (3)	0.4599 (2)	0.6797 (4)	0.0464 (10)	
C9	0.4730 (3)	0.4824 (2)	0.7582 (4)	0.0463 (10)	
H9	0.4756	0.5325	0.8007	0.056*	
C10	0.3900 (3)	0.4308 (2)	0.7768 (4)	0.0409 (9)	
C11	0.3054 (3)	0.4576 (2)	0.8616 (4)	0.0428 (9)	
H11	0.3369	0.4829	0.9501	0.051*	
C12	0.2446 (3)	0.3864 (2)	0.9034 (4)	0.0390 (9)	
C13	0.2453 (3)	0.3168 (2)	0.8286 (4)	0.0394 (9)	
C14	0.1773 (3)	0.3946 (2)	1.0137 (4)	0.0428 (9)	
C15	0.1070 (4)	0.4882 (3)	1.1673 (5)	0.0741 (14)	
H15A	0.1343	0.4669	1.2601	0.089*	
H15B	0.0400	0.4652	1.1401	0.089*	
C16	0.0999 (5)	0.5766 (3)	1.1742 (6)	0.097 (2)	
H16A	0.0550	0.5913	1.2435	0.146*	
H16B	0.0736	0.5968	1.0814	0.146*	
H16C	0.1667	0.5984	1.2020	0.146*	
C17	0.2379 (3)	0.5190 (2)	0.7770 (4)	0.0426 (9)	
C18	0.1791 (3)	0.4979 (2)	0.6511 (5)	0.0516 (10)	
H18	0.1789	0.4454	0.6199	0.062*	
C19	0.1203 (3)	0.5538 (3)	0.5709 (5)	0.0582 (12)	
H19	0.0813	0.5392	0.4858	0.070*	
C20	0.1202 (3)	0.6312 (3)	0.6184 (5)	0.0568 (12)	
C21	0.1771 (4)	0.6536 (3)	0.7419 (5)	0.0659 (13)	
H21	0.1764	0.7061	0.7731	0.079*	
C22	0.2362 (3)	0.5972 (3)	0.8208 (5)	0.0588 (12)	
H22	0.2756	0.6124	0.9052	0.071*	
C23	0.6286 (3)	0.5869 (3)	0.6996 (5)	0.0624 (12)	
H23A	0.6879	0.6138	0.6725	0.094*	
H23B	0.6302	0.5878	0.8023	0.094*	
H23C	0.5680	0.6133	0.6566	0.094*	

### Atomic displacement parameters (Å^2^)

**Table:**

	*U*^11^	*U*^22^	*U*^33^	*U*^12^	*U*^13^	*U*^23^
Br1	0.0945 (4)	0.0850 (5)	0.0981 (5)	0.0486 (3)	0.0437 (3)	0.0394 (3)
O1	0.0453 (14)	0.0431 (16)	0.0537 (18)	−0.0112 (12)	0.0179 (12)	−0.0067 (13)
O2	0.0601 (17)	0.0524 (19)	0.0521 (18)	−0.0043 (14)	0.0187 (13)	0.0031 (14)
O3	0.0770 (19)	0.0445 (17)	0.0495 (18)	0.0043 (14)	0.0229 (14)	−0.0045 (14)
O4	0.0519 (17)	0.058 (2)	0.083 (2)	−0.0210 (15)	0.0179 (14)	−0.0057 (17)
N1	0.058 (2)	0.046 (2)	0.062 (3)	−0.0103 (19)	0.0252 (19)	−0.0082 (19)
C1	0.0367 (19)	0.042 (2)	0.041 (2)	−0.0061 (17)	0.0063 (16)	0.0040 (18)
C2	0.0375 (19)	0.042 (2)	0.040 (2)	0.0000 (17)	0.0032 (15)	0.0032 (18)
C3	0.043 (2)	0.043 (2)	0.047 (2)	−0.0030 (19)	0.0062 (17)	−0.0026 (19)
C4	0.059 (3)	0.050 (3)	0.052 (3)	0.004 (2)	0.012 (2)	−0.006 (2)
C5	0.049 (3)	0.069 (3)	0.068 (3)	0.001 (2)	0.019 (2)	−0.008 (3)
C6	0.046 (2)	0.063 (3)	0.068 (3)	−0.007 (2)	0.018 (2)	−0.002 (2)
C7	0.036 (2)	0.047 (2)	0.048 (2)	−0.0049 (18)	0.0054 (16)	0.0043 (19)
C8	0.041 (2)	0.047 (2)	0.051 (3)	−0.0103 (18)	0.0025 (17)	0.001 (2)
C9	0.045 (2)	0.040 (2)	0.054 (3)	−0.0053 (18)	0.0043 (18)	−0.0024 (19)
C10	0.039 (2)	0.041 (2)	0.041 (2)	−0.0011 (17)	0.0025 (16)	0.0002 (18)
C11	0.049 (2)	0.041 (2)	0.039 (2)	−0.0021 (18)	0.0034 (16)	−0.0040 (18)
C12	0.043 (2)	0.036 (2)	0.039 (2)	0.0007 (17)	0.0069 (16)	0.0008 (17)
C13	0.0392 (19)	0.037 (2)	0.043 (2)	−0.0039 (17)	0.0095 (16)	0.0037 (18)
C14	0.044 (2)	0.044 (2)	0.040 (2)	0.0026 (19)	0.0023 (17)	0.0041 (19)
C15	0.102 (4)	0.071 (4)	0.055 (3)	0.018 (3)	0.029 (3)	−0.008 (3)
C16	0.151 (6)	0.073 (4)	0.073 (4)	0.044 (4)	0.037 (4)	−0.005 (3)
C17	0.046 (2)	0.035 (2)	0.048 (2)	−0.0047 (17)	0.0116 (17)	−0.0024 (18)
C18	0.060 (2)	0.038 (2)	0.056 (3)	0.001 (2)	0.007 (2)	0.001 (2)
C19	0.056 (3)	0.064 (3)	0.054 (3)	0.002 (2)	0.007 (2)	0.010 (2)
C20	0.056 (3)	0.050 (3)	0.070 (3)	0.018 (2)	0.031 (2)	0.016 (2)
C21	0.093 (3)	0.038 (3)	0.072 (3)	0.014 (2)	0.032 (3)	0.000 (2)
C22	0.077 (3)	0.042 (3)	0.058 (3)	0.000 (2)	0.012 (2)	−0.008 (2)
C23	0.061 (3)	0.050 (3)	0.074 (3)	−0.016 (2)	−0.001 (2)	0.008 (2)

### Geometric parameters (Å, º)

**Table:**

Br1—C20	1.903 (4)	C9—H9	0.9300
O1—C13	1.362 (4)	C10—C11	1.508 (5)
O1—C1	1.400 (4)	C11—C12	1.517 (5)
O2—C14	1.218 (4)	C11—C17	1.524 (5)
O3—C14	1.346 (5)	C11—H11	0.9800
O3—C15	1.450 (5)	C12—C13	1.364 (5)
O4—C8	1.369 (4)	C12—C14	1.442 (5)
O4—C23	1.419 (5)	C15—C16	1.489 (7)
N1—C13	1.338 (5)	C15—H15A	0.9700
N1—H2	0.876 (10)	C15—H15B	0.9700
N1—H1	0.878 (10)	C16—H16A	0.9600
C1—C10	1.359 (5)	C16—H16B	0.9600
C1—C2	1.417 (5)	C16—H16C	0.9600
C2—C3	1.413 (5)	C17—C22	1.378 (5)
C2—C7	1.420 (5)	C17—C18	1.380 (5)
C3—C4	1.366 (5)	C18—C19	1.382 (6)
C3—H3	0.9300	C18—H18	0.9300
C4—C5	1.397 (6)	C19—C20	1.375 (6)
C4—H4	0.9300	C19—H19	0.9300
C5—C6	1.355 (6)	C20—C21	1.356 (6)
C5—H5	0.9300	C21—C22	1.384 (6)
C6—C7	1.418 (6)	C21—H21	0.9300
C6—H6	0.9300	C22—H22	0.9300
C7—C8	1.430 (6)	C23—H23A	0.9600
C8—C9	1.354 (6)	C23—H23B	0.9600
C9—C10	1.421 (5)	C23—H23C	0.9600
			
C13—O1—C1	117.9 (3)	C14—C12—C11	119.7 (3)
C14—O3—C15	117.4 (3)	N1—C13—O1	109.9 (3)
C8—O4—C23	117.3 (3)	N1—C13—C12	127.2 (4)
C13—N1—H2	114 (4)	O1—C13—C12	122.9 (3)
C13—N1—H1	115 (3)	O2—C14—O3	121.9 (4)
H2—N1—H1	125 (5)	O2—C14—C12	126.8 (4)
C10—C1—O1	122.1 (3)	O3—C14—C12	111.2 (4)
C10—C1—C2	123.0 (3)	O3—C15—C16	106.5 (4)
O1—C1—C2	114.9 (3)	O3—C15—H15A	110.4
C1—C2—C3	122.9 (3)	C16—C15—H15A	110.4
C1—C2—C7	117.8 (3)	O3—C15—H15B	110.4
C3—C2—C7	119.4 (3)	C16—C15—H15B	110.4
C4—C3—C2	120.2 (4)	H15A—C15—H15B	108.6
C4—C3—H3	119.9	C15—C16—H16A	109.5
C2—C3—H3	119.9	C15—C16—H16B	109.5
C3—C4—C5	120.6 (4)	H16A—C16—H16B	109.5
C3—C4—H4	119.7	C15—C16—H16C	109.5
C5—C4—H4	119.7	H16A—C16—H16C	109.5
C6—C5—C4	120.5 (4)	H16B—C16—H16C	109.5
C6—C5—H5	119.7	C22—C17—C18	118.3 (4)
C4—C5—H5	119.7	C22—C17—C11	121.3 (4)
C5—C6—C7	121.1 (4)	C18—C17—C11	120.4 (3)
C5—C6—H6	119.4	C19—C18—C17	120.8 (4)
C7—C6—H6	119.4	C19—C18—H18	119.6
C6—C7—C2	118.1 (4)	C17—C18—H18	119.6
C6—C7—C8	123.1 (4)	C18—C19—C20	119.3 (4)
C2—C7—C8	118.8 (3)	C18—C19—H19	120.4
C9—C8—O4	125.0 (4)	C20—C19—H19	120.4
C9—C8—C7	120.8 (4)	C21—C20—C19	121.2 (4)
O4—C8—C7	114.3 (3)	C21—C20—Br1	119.8 (4)
C8—C9—C10	121.1 (4)	C19—C20—Br1	119.0 (4)
C8—C9—H9	119.5	C20—C21—C22	119.0 (4)
C10—C9—H9	119.5	C20—C21—H21	120.5
C1—C10—C9	118.5 (4)	C22—C21—H21	120.5
C1—C10—C11	121.0 (3)	C17—C22—C21	121.4 (4)
C9—C10—C11	120.4 (3)	C17—C22—H22	119.3
C10—C11—C12	110.1 (3)	C21—C22—H22	119.3
C10—C11—C17	110.4 (3)	O4—C23—H23A	109.5
C12—C11—C17	112.1 (3)	O4—C23—H23B	109.5
C10—C11—H11	108.0	H23A—C23—H23B	109.5
C12—C11—H11	108.0	O4—C23—H23C	109.5
C17—C11—H11	108.0	H23A—C23—H23C	109.5
C13—C12—C14	119.5 (4)	H23B—C23—H23C	109.5
C13—C12—C11	120.6 (3)		
			
C13—O1—C1—C10	−17.1 (5)	C1—C10—C11—C17	−105.9 (4)
C13—O1—C1—C2	162.4 (3)	C9—C10—C11—C17	72.4 (4)
C10—C1—C2—C3	178.4 (3)	C10—C11—C12—C13	−21.7 (5)
O1—C1—C2—C3	−1.0 (5)	C17—C11—C12—C13	101.7 (4)
C10—C1—C2—C7	−1.7 (5)	C10—C11—C12—C14	164.5 (3)
O1—C1—C2—C7	178.8 (3)	C17—C11—C12—C14	−72.1 (4)
C1—C2—C3—C4	−179.5 (4)	C1—O1—C13—N1	−167.7 (3)
C7—C2—C3—C4	0.7 (5)	C1—O1—C13—C12	13.7 (5)
C2—C3—C4—C5	−1.0 (6)	C14—C12—C13—N1	2.2 (6)
C3—C4—C5—C6	0.2 (7)	C11—C12—C13—N1	−171.6 (4)
C4—C5—C6—C7	0.8 (7)	C14—C12—C13—O1	−179.5 (3)
C5—C6—C7—C2	−1.0 (6)	C11—C12—C13—O1	6.8 (5)
C5—C6—C7—C8	179.8 (4)	C15—O3—C14—O2	−3.3 (6)
C1—C2—C7—C6	−179.5 (4)	C15—O3—C14—C12	177.5 (3)
C3—C2—C7—C6	0.3 (5)	C13—C12—C14—O2	10.4 (6)
C1—C2—C7—C8	−0.4 (5)	C11—C12—C14—O2	−175.7 (3)
C3—C2—C7—C8	179.5 (3)	C13—C12—C14—O3	−170.4 (3)
C23—O4—C8—C9	−4.4 (6)	C11—C12—C14—O3	3.4 (5)
C23—O4—C8—C7	174.5 (3)	C14—O3—C15—C16	−166.9 (4)
C6—C7—C8—C9	−178.7 (4)	C10—C11—C17—C22	−111.9 (4)
C2—C7—C8—C9	2.1 (6)	C12—C11—C17—C22	125.0 (4)
C6—C7—C8—O4	2.2 (6)	C10—C11—C17—C18	66.0 (5)
C2—C7—C8—O4	−176.9 (3)	C12—C11—C17—C18	−57.2 (5)
O4—C8—C9—C10	177.0 (3)	C22—C17—C18—C19	0.2 (6)
C7—C8—C9—C10	−1.9 (6)	C11—C17—C18—C19	−177.7 (4)
O1—C1—C10—C9	−178.5 (3)	C17—C18—C19—C20	−0.5 (6)
C2—C1—C10—C9	2.1 (5)	C18—C19—C20—C21	0.4 (7)
O1—C1—C10—C11	−0.2 (5)	C18—C19—C20—Br1	178.9 (3)
C2—C1—C10—C11	−179.6 (3)	C19—C20—C21—C22	0.1 (7)
C8—C9—C10—C1	−0.2 (6)	Br1—C20—C21—C22	−178.4 (3)
C8—C9—C10—C11	−178.5 (4)	C18—C17—C22—C21	0.2 (7)
C1—C10—C11—C12	18.4 (5)	C11—C17—C22—C21	178.1 (4)
C9—C10—C11—C12	−163.3 (3)	C20—C21—C22—C17	−0.4 (7)

### Hydrogen-bond geometry (Å, º)

Cg1, Cg2 and Cg3 are the centroids of the C1,C2,C7–C10, C17–C22 and C2–C7 rings, respectively.

**Table:**

*D*—H···*A*	*D*—H	H···*A*	*D*···*A*	*D*—H···*A*
N1—H2···O2	0.88 (1)	2.09 (5)	2.744 (5)	131 (5)
N1—H1···O2^i^	0.88 (1)	2.22 (2)	3.075 (5)	163 (3)
N1—H2···Br1^ii^	0.88 (4)	2.76 (4)	3.547 (4)	149 (5)
C4—H4···*Cg*1^i^	0.93	2.90	3.673 (5)	142
C6—H6···*Cg*2^iii^	0.93	2.98	3.743 (5)	140
C23—H23*C*···*Cg*3^iii^	0.96	2.70	3.593 (5)	154

Symmetry codes: (i) *x*, −*y*+1/2, *z*−1/2; (ii) −*x*, *y*−1/2, −*z*+3/2; (iii) −*x*+1, −*y*+1, −*z*+1.
